# Biological screening for congenital toxoplasmosis in newborns from Jataí, Goiás, Brazil: a cross-sectional study

**DOI:** 10.1590/1980-220X-REEUSP-2023-0408en

**Published:** 2024-07-15

**Authors:** Gabriela Katrinny Avelar Oliveira, Stéfanne Rodrigues Rezende Ferreira, Vanessa Oliveira Lopes de Moura, Victor da Silva Siqueira, Thaís Santos Anjo Reis, Vanessa Bridi, Ludimila Paula Vaz Cardoso, Hanstter Hallison Alves Rezende

**Affiliations:** 1 Universidade Federal de Jataí, Instituto de Ciências da Saúde, Programa de Pós-Graduação em Ciências Aplicadas à Saúde, Jataí, GO, Brazil.

**Keywords:** Toxoplasma, Toxoplasmosis, Congenital, Neonatal Screening, Toxoplasma, Toxoplasmosis Congénita, Tamizaje Neonatal

## Abstract

**Objective:**

To conduct a serological screening for toxoplasmosis in the heel prick test and to evaluate its epidemiological aspects in newborns and postpartum women in Jataí, Goiás.

**Method:**

Cross-sectional epidemiological study for the biological screening of newborns in Jataí, Goiás.

**Results:**

The study participants amounted to 228 newborns, whose samples were collected between the third and seventh day of life. IgG antibodies against Toxoplasma gondii were detected in 40.79% (93/228) of the samples; out of these, 23.6% (22/93) had high IgG antibody titers, leading to the collection of two other peripheral blood samples and the detection of a decrease in these titers.

**Conclusion:**

The findings show the importance of strengthening actions in primary health care to prevent infection and training health professionals in this area to equip them with information regarding cases of reinfection and reactivation of infection in pregnant women, minimizing risks for babies.

## RESUMEN

**Objetivo:**

Realizar un cribado serológico de la toxoplasmosis en la prueba del talón y evaluar sus aspectos epidemiológicos en los recién nacidos y puérperas en Jataí, Goiás.

**Método:**

Estudio epidemiológico transversal para el cribado biológico de los recién nacidos en Jataí, Goiás.

**Resultados:**

Participaron en el estudio 228 recién nacidos, cuyas muestras fueron recolectadas entre el tercer y séptimo día de vida. Se detectaron anticuerpos IgG contra *Toxoplasma gondii* en el 40,79% (93/228) de las muestras; de ellos el 23,6% (22/93) presentaban títulos elevados de anticuerpos IgG y de ellos se recogieron otras dos muestras de sangre periférica; se detectó una disminución de estos títulos.

**Conclusión:**

Los hallazgos muestran la importancia de fortalecer las acciones en la atención primaria de salud para prevenir la infección y capacitar a los profesionales de la salud en esta área para dotarlos de información sobre los casos de reinfección y reactivación de la infección en mujeres embarazadas, minimizando los riesgos para los bebés.

## INTRODUCTION

Infectious diseases during pregnancy are one of the major problems facing public health. These include toxoplasmosis, rubella, hepatitis B and C, syphilis, human immunodeficiency virus (HIV), among others, which can be transplacentally transmitted from mother to child at birth, possibly leading to miscarriage and premature birth, or during breastfeeding^(1,2)^.

Prenatal care must be based on scientific evidence, respecting the individualities of each pregnant woman, to minimize the risks that may arise during pregnancy due to infectious diseases^(3)^. The National Neonatal Screening Program (*Programa Nacional de Triagem Neonatal* – PNTN) was implemented in Brazil through ordinance no. 822, dated June 6, 2001, in the Unified Health System (*Sistema* Único *de Saúde* – SUS). This program consists of a set of actions aimed at the prevention and early identification and treatment of children who may have metabolic, genetic, enzymatic and endocrinological diseases^(4)^.

The main actions of the PNTN include conducting the Guthrie test, known as the heel prick test, which enables an early diagnosis of six diseases: sickle cell anemia, phenylketonuria, congenital hypothyroidism, biotinidase deficiency, cystic fibrosis, and congenital adrenal hyperplasia. These may not manifest symptoms in early childhood, but they cause several child health problems. In 2021, the PNTN was improved to establish a list of diseases to be tracked through the heel prick tests, which will be gradually implemented in five stages^(5)^. The test must be conducted between the 3rd and 5th day of the newborn’s life. Carrying it out before 48 hours of life is not recommended and, if it has not been done by the seventh day of life, it should be performed within 30 days after birth^(6)^.

In gestational risk, infection by *Toxoplasma gondii* is highlighted; this is the etiological agent of toxoplasmosis, an infection that is self-limited in healthy people, but which can present several risks for the gestational period^(7)^. Toxoplasmosis is common in countries with a tropical climate and can be acquired mainly through inadequate eating and hand and food hygiene habits, in addition to the congenital form, when transmitted from mother to child^(8,9)^.

Toxoplasmosis by primary infection is the most common; it is the individual’s first contact with the parasite. It is usually asymptomatic in immunocompetent individuals; however, after acute infection, the parasite remains in chronic form in tissue cysts, and can manifest at any time in an individual’s life^(10)^.

Toxoplasmosis is notorious when manifested by women of childbearing age, due to the risk it presents to the fetus. As the gestational process can lead to immunological deficiency in some women, there is a risk of cyst reactivation, generating thus a risk of fetal compromise, vertical transmission, local and systemic manifestations in the baby, spontaneous abortion, and fetal death^(11)^.

In Brazil, around 60% of the adult population is estimated to have been exposed to *T. gondii*, due to its territorial extension and to sociocultural differences. Although there are different prevalence rates per region, high values are always identified; this may be closely linked to eating habits and environmental conditions^(8,11)^. The rate of women of childbearing age exposed to this parasite and presenting chronic infection is 83%^(7)^.

As for congenital toxoplasmosis worldwide, it is estimated that, out of ten thousand live births, 1 to 10 children will be infected by *T. gondii* ^(8,11)^. In the USA, it is estimated that around four hundred children are born with congenital toxoplasmosis per year, reaching 4,000 for every 10,000 live births^(7)^. South America has a high incidence rate; in Brazil, the Ministry of Health’s (MH) estimate is that, out of ten thousand live births, 3 to 20 present congenital toxoplasmosis and may show the signs and symptoms of this infection throughout their lives^(6)^.

The highest prevalence rates of gestational infections are identified in low-income women, which highlights the challenges for basic care and for strengthening prevention, diagnosis, and early treatment actions in this population^(12)^. When pregnant women are exposed to *T. gondii*, whether in an acute infection or in the reactivation of a chronic infection, have a high risk of transplacental transmission, which can trigger spontaneous abortion and fetal sequelae, such as neurological and ocular pathologies; these may also manifest through changes in life adult.

Health professionals responsible for prenatal care have a crucial role in disseminating quality information and guidance regarding the disease, aiming to minimize risk factors for pregnant women. These professionals must also request laboratory tests during pregnancy, in addition to informing pregnant women of their importance. These tests enable identifying the parasite and so that treatment can be started, aiming to reduce the risk of transplacental transmission, in addition to monitoring the newborn through the heel prick test.

Studies are therefore needed to demonstrate the clinical, laboratory, and epidemiological profile of congenital toxoplasmosis in newborns in order to improve the quality of prevention, diagnosis, and treatment actions. The objective of this study is to perform a serological screening for toxoplasmosis through the heel prick test and evaluate the epidemiological aspects of newborns and puerperal women in the municipality of Jataí, in the state of Goiás, Brazil.

## METHOD

### Type of Study

This is a cross-sectional epidemiological study.

### Local

The study was conducted in the municipality of Jataí, Goiás, Brazil, in the only place where neonatal screening of newborns is performed, and in the Laboratory of Clinical Biochemistry and Body Fluids of the Biomedicine course of Universidade Federal de Jataí.

### Selection Criteria

The samples were selected by convenience and comprised parents and/or guardians who took their newborns to the municipal health center for the heel prick test between the third and seventh day of life. They were informed by the researchers about the study and invited to participate. After they read and signed the Informed Consent Form (ICF), the filter paper sample was collected. The researchers are biomedical scientists and nurses.

When the ELISA analysis showed a positive IgM result or high IgG antibody titers, the newborns’ parents and/or guardians were notified and authorization was requested for a second collection of a peripheral blood sample from the baby and the mother to carry out new ELISA laboratory tests so that they could be followed and monitored by the researchers, aiming at the identification of congenital toxoplasmosis. This collection was carried out at the participants’ homes.

### Sample Definition

This study’s participants amounted to 228 newborn babies. The average number of heel prick tests conducted in the municipality of Jataí in the period before the COVID-19 pandemic was calculated. In 2017, 1,216 heel prick test collections were carried out; in 2018, 1,327 collections; in 2019, 1,248 collections; and in 2020, 1,104 collections. From this, the sample was calculated, indicating that at least 205 heel prick test samples were required to perform this study, which could present a 6.25% margin of error^(13)^.

### Biological Sample Collection on Filter Paper

The newborn blood samples were collected on filter paper between September 2021 and May 2022.

The newborns whose parents agreed to participate had their blood sample collected on Whatman n. 1 filter paper, measuring 3 cm. The samples were placed on shelves in a horizontal position, so that there was no contact with the others, until the paper was dry (room temperature). They were then kept in suitable envelopes and identified using a standardized stamp. The material was transported to the Clinical Biochemistry and Body Liquids Laboratory for serological analysis, and packaging and transportation followed the recommendations of the Brazilian Ministry of Health’s Neonatal Screening Technical Manual^(6)^.

### Standardization of the Enzyme Immunoassay Technique on Filter Paper

The technique was standardized and titers were defined according to the guidelines provided by the manufacturer of the commercial kit *SERION ELISA classic-Toxoplasma gondii IgG/IgM* ^®^. After evaluating the results as determined by the Quality Control Certificate, OD values above 1.4494 in the analysis of absorbance were defined as a high titer of IgG antibodies.

### Sample Preparation and Serological Diagnosis

The filter paper samples were prepared the day before the test with personal protective equipment (PPE), an individual punch, to cut out circles of 3 to 5 mm, eppendorf tube, and pipette.

For IgM measurement, the circle was eluted in a solution of RF-absorbent ¼; therefore, 1 circle of 3 to 5 µL + 100 µL of RF-absorbent + 400 µL of sample buffer were utilized. For the detection of IgG, the sample was eluted in 100 µL of sample buffer. For both dosages, the filter paper was completely covered with the buffer and could not float inside the tube. They were then incubated overnight in the refrigerator at 2 to 8^º^C.

The samples were then submitted to serological examination and the result enabled determining the presence of *T. gondii* and the IgM and IgG immunoglobulins.

### Monitoring Newborns with High Antibody Titers

Babies presenting high IgG antibody titers (OD ≥ 1.4494) in the absorbance analysis were monitored to verify possible manifestations of congenital toxoplasmosis infection, as well as their laboratory data. A total of twenty-two babies were monitored.

A second, venous collection was carried out with a safety margin of three months from the initial collection on filter paper, taking into account the half-life of IgM and IgG antibodies, thus enabling observing the baby’s antibodies, and not the mother’s. A 5 mL sample of peripheral blood was collected from the mother and baby, placed in tubes to obtain serum, and transported to the Laboratory of Clinical Biochemistry and Body Fluids at Universidade Federal de Jataí.

After about a year, the monitored babies underwent a new blood collection to check whether the antibody titers continued to decrease or had become non-reactive.

Authorization for a third venous collection from eighteen children was obtained. The parents and guardians were then instructed about the clinical manifestations of congenital and acquired toxoplasmosis, as well as reference services in the municipality of Jataí for child health care.

### Questionnaire Application

When the baby’s second blood sample was collected, the mother’s blood was also collected. Furthermore, a socioeconomic and behavioral questionnaire constructed by the researchers was applied to twenty-two mothers of babies monitored due to high antibody titers to collect relevant information about the profile of the population. Mothers who agreed to participate in the study were interviewed in an appropriate location, following all safety recommendations and health protocols proposed by the health service. The data were used for epidemiological analysis as risk factors for contamination/transmission of *T. gondii*. The newborn was then assessed to check for possible changes due to toxoplasmosis for follow-up and the guiding document for home visit was completed.

### Data Analysis and Treatment

The data were analyzed using the Microsoft Excel^®^ 2021 program and presented with descriptive analytical statistics, highlighting the prevalence of pregnant women with toxoplasmosis whose newborn might present a congenital infection.

### Ethical Aspects

The project was approved by the Research Ethics Committee (REC) in opinion number 4,925,393, dated 2021. All participants signed the ICF to participate and all the guidelines contained in Resolutions n. 466/2012 and 510/2016, dealing with research involving human beings, were followed.

## RESULTS

### Serological Screening on Filter Paper

**Figure 1:**
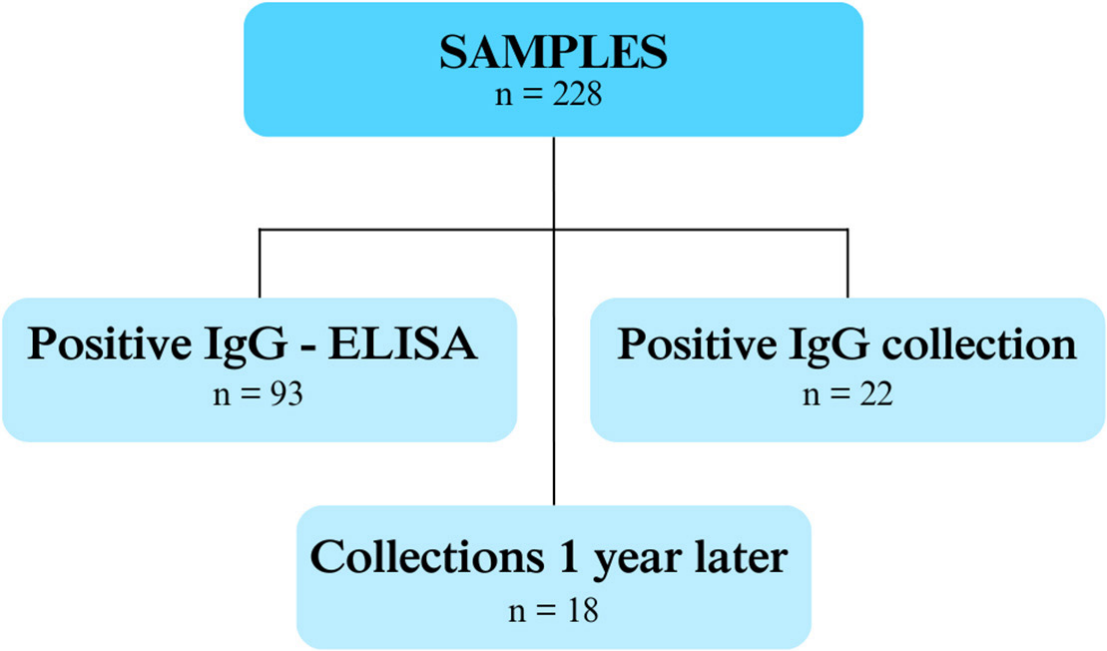
Distribution of the number of samples.

Out of 228 samples, IgG anti- *T. gondii* antibodies were detected in 40.8% (93/228) and IgM was not identified by the ELISA test. Out of the samples which were positive for the *T. gondii* IgG antibody, 23.6% (22/93) had high antibody values, being referred for a new collection of baby and mother peripheral blood samples. Figure 1 shows the number of samples evaluated in this study.

The babies’ serologies in the initial, second and third collections, as well as that of their respective mothers, were compared, as shown in Table 1.

**Table 1:** Comparison of Immunoglobulin G serologies collected between September 2021 and May 2022 and in April 2023 – Jataí, GO, Brazil, 2023.

Code	1st collection* Filter Paper		2nd collection* Peripheral blood				3rd collection*** Peripheral blood	
	Child		Mother		Child		Child	
002	1.491	POS	2.175	POS	0.241	NEG	0.023	NEG
003	1.577	POS	2.023	POS	0.246	NEG	0.028	NEG
006	1617	POS	2.258	POS	0.210	NEG	0.008	NEG
056	1.427	POS	2.214	POS	0.162	NEG	0.056	NEG
091	1.451	POS	2.124	POS	0.204	NEG	0.014	NEG
151	1.499	POS	2.395	POS	0.175	NEG	0.043	NEG
174	1.633	POS	3.020	POS	0.243	NEG	0.025	NEG
177	1.654	POS	2.731	POS	0.305	NEG	0.087	NEG
179	1.464	POS	2.086	POS	0.321	NEG	0.103	NEG
182	1.488	POS	2.149	POS	0.297	NEG	0.079	NEG
183	1.553	POS	2.174	POS	0.181	NEG	0.037	NEG
186	1.642	POS	2.985	POS	0.249	NEG	0.031	NEG
208	1.862	POS	2.325	POS	0.229	NEG	0.011	NEG
209	2.148	POS	3.122	POS	0.235	NEG	NR	NEG
210	2.240	POS	2.996	POS	0.222	NEG	0.017	NEG
211	1.951	POS	2.827	POS	0.292	NEG	NR	NEG
214	1.582	POS	1.795	POS	0.195	NEG	0.004	NEG
223	1.458	POS	1.637	POS	0.302	NEG	0.074	NEG
224	2.073	POS	2.894	POS	0.241	NEG	NR	NEG
225	1.875	POS	3.061	POS	0.288	NEG	NR	NEG
226	1.890	POS	3.038	POS	0.232	NEG	0.023	NEG
228	1.862	POS	3.073	POS	0.323	NEG	0.084	NEG

*Filter paper collection, carried out from 3 to 5 days of age. ** Collected from 3 to 4 months of age. *** Collected from 12 to 18 months of age. POS: positive, NEG: negative.

### Sociodemographic Profile

A mean age of 24.3 years was verified (18 years – 30 years). Most participants were married, 72.7% (16/22) and all lived in the urban area of the municipality of Jataí–GO. The most frequent level of education was complete secondary education: 81.8% (18/22). The findings on the parents’ basic sanitation and lifestyle habits can be seen in Chart 1.

**Chart 1:** Sociodemographic profile of the study participants’ parents and guardians – Jataí, GO, Brazil.

Variables		N. (n = 22)	Percentage (%)
Origin of drinking water	Public network	22	100
Sewage outlet	Public network	22	100
Disposal of household waste	Public collection	22	100
Vegetable garden at home	Yes	2	9.1
	No	22	90.9
Person preparing food	Mother	17	77.28
	Others	5	22.72
Pets at home	Cat	0	0
	Dog	6	27.8
Behavioral and eating habits	Handles earth or sand	2	9.1
	Eats meat daily	22	100
	Consumes raw or undercooked meat sporadically	5	22.7

The sociodemographic profile showed that women had a mean of two pregnancies. Only one interviewee reported a case of miscarriage without a defined cause. The mean gestational age at delivery was 39 weeks (37 – 41 weeks). All interviewees received prenatal care through SUS in Basic Health Units close to their home and all women underwent both stages of prenatal screening, as verified on their prenatal care card. The results were reactive for anti *T. gondii* IgG and none had reactive IgM antibodies. All had normal-risk pregnancies.

## DISCUSSION

The sample analysis through the ELISA method demonstrated a significant decrease in the values of the children’s IgG antibodies, which allows inferring that the antibodies of the first collection came from the mothers and passively crossed the transplacental barrier, starting to circulate in the baby’s blood^(8,9)^.

The high prevalence of chronically infected women in the region, as verified by the high rates of babies with circulating *T. gondii* antibodies, as it increases the risks of infection reactivation in women and congenital transmission^(11)^. Furthermore, risk of infection may be increased in women of childbearing age, due to a high circulation of the parasite in the municipality^(13)^.

Compared to other Brazilian regions, the prevalence of gestational toxoplasmosis in the municipality seems to corroborate findings in Goiânia, 41.8%^(14,15)^, in Santa Catarina, 47%^(15)^, in Sergipe, with approximately 50%^(16)^, in São Paulo, 54.91%^(17)^. The municipality has a lower prevalence when compared to clinical findings in Amapá, with 73.43% of infected pregnant women^(18)^, and a higher prevalence compared to the state of Rio Grande do Norte, with a prevalence of 24.4%^(19)^. These data reinforce that Brazil has high rates of *T. gondii* infection, particularly in women of childbearing age, which validates the importance of health surveillance actions to minimize the risk of congenital infections.

No cases of congenital toxoplasmosis were found in this study, which, despite being a serious form of the disease, is less common. In a cohort study with women screened during early pregnancy, the rate of vertical transmission was found to be < 5% when infection sets in 3 months before the pregnancy or in the first months, reaching up to 71% if there is an acute infection after 37 weeks of pregnancy^(20)^. In countries like the United States, the incidence of congenital toxoplasmosis from 2006 to 2014 was found to be only 0.23% per ten thousand live births^(21)^.

Prenatal care provided by SUS, which was chosen by the women interviewed in this study, presents regional inequalities, but has a high coverage throughout the country. In the Center-West region, in addition to high coverage, monitoring is started early, allowing for better assessment and more time for important information about pregnancy to be passed on^(22)^.

Despite the high adherence, adequate prenatal care rates are low when taking into consideration those including a minimum of six prenatal consultations, all necessary laboratory tests, and at least three follow-up ultrasounds, in addition to reliable guidance based on clearly and objectively communicated technical and scientific knowledge to be wholly understood by patients; failure to comply with these requirements worsens the health outcomes of pregnant women^(23)^.

Regarding guidance from health professionals, 86.4% (19/22) reported having been informed about toxoplasmosis when the test results were delivered, but regarded orientation as superficial, as important information regarding the finding of anti-IgG antibodies for *T. gondii* was not provided. Other 13.63% (3/22) received no guidance on the prenatal screening’s findings.

As in other studies’ findings, the health professionals did not present an adequate knowledge of toxoplasmosis, especially of its congenital form, cases of reinfection, and *T. gondii* infection reactivation, as well as on the risks of transmission, prophylactic measures, and clinical manifestations in babies^(12,24)^. Furthermore, many professionals consider the appearance of IgG antibodies in prenatal exams to be a protective factor for pregnant women. They are not provided with important information on the susceptibility to reinfection and reactivation, effective prophylactic measures, and signs and symptoms of the disease^(12)^.

The health professionals’ lack of knowledge leads them to neglect cases of gestational toxoplasmosis, thus increasing the risk of transplacental transmission and congenital toxoplasmosis, exposing women and children to other risks of this disease’s manifestations^(11)^. When health professionals correctly identify acute *T. gondii* infections and starts treatment for pregnant women early and correctly, the chances of vertical transmission are drastically reduced, especially if started before the third week after seroconversion^(25)^.

In countries such as Austria and France, a decrease in the prevalence of toxoplasmosis and the incidence of congenital infections was reduced through the implementation of prenatal screening, favoring the early start of treatment, and the enhancement of health education actions^(26)^. In Brazil, prenatal screening is regulated by the Ministry of Health, oriented by protocols and manuals, aiming at early detection of previous infections during pregnancy^(27)^. In the State of Goiás, all pregnant women who undergo prenatal care at SUS are submitted to prenatal screening, which includes the toxoplasmosis test^(28)^. Therefore, it is extremely important for health professionals to know how to interpret exams and provide correct guidance, since women’s access to exams is guaranteed^(12,27)^.

The limitations of this study were mainly related to the difficulty in approaching families for the baby monitoring stages, as many were not aware that this procedure was necessary due to a lack of knowledge of this disease and little or incorrect orientation from health services.

This study contributes particularly to an understanding of the epidemiological profile of the municipality, in addition to collecting information on the quality of guidance on toxoplasmosis provided to families by health professionals. Professional updating is required for the staff to recognize the importance of this disease, care during the pregnancy cycle, correct exam interpretation, and the existence of cases such as reinfection and infection reactivation.

## CONCLUSION

Neonatal screening for toxoplasmosis was verified to allow the detection of IgG antibodies, which were high in the municipality, as ninety-three cases were found, presenting thus a considerable rate of chronic infections in women of childbearing age. These findings demonstrate the importance of strengthening primary health care actions to prevent this infection, as well as training health professionals working in this area to equip them with information regarding cases of reinfection and infection reactivation in pregnant women, in order to minimize the risks for babies.
