# Supplementary material for: Biological screening for congenital toxoplasmosis in newborns from Jataí, Goiás, Brazil: a cross-sectional study
Source: Rev Esc Enferm USP. 2024 Jul 15;58:e20230408. doi: 10.1590/1980-220X-REEUSP-2023-0408en (PMC11268135; doi:10.1590/1980-220X-REEUSP-2023-0408en)
Supplement: Translation

# Translation: Triagem biológica de toxoplasmose congênita em recém-nascidos de Jataí, Goiás: um estudo transversal

**DOI:** 10.1590/1980-220X-REEUSP-2023-0408pt

**Authors:** Gabriela Katrinny Avelar Oliveira, Stéfanne Rodrigues Rezende Ferreira, Vanessa Oliveira Lopes de Moura, Victor da Silva Siqueira, Thaís Santos Anjo Reis, Vanessa Bridi, Ludimila Paula Vaz Cardoso, Hanstter Hallison Alves Rezende

**Affiliations:** 1 Universidade Federal de Jataí, Instituto de Ciências da Saúde, Programa de Pós-Graduação em Ciências Aplicadas à Saúde, Jataí, GO, Brasil.

**Keywords:** Toxoplasma, Toxoplasmose Congênita, Triagem Neonatal

## Abstract

**Objetivo:**

Realizar a triagem sorológica da toxoplasmose no teste do pezinho e avaliar os aspectos epidemiológicos em recém-nascidos e puérperas de Jataí, Goiás.

**Método:**

Estudo epidemiológico transversal para a triagem biológica de recém-nascidos em Jataí, Goiás.

**Resultados:**

Participaram desta pesquisa 228 recém-nascidos, sendo coletadas amostras entre o terceiro e o sétimo dia de vida. Foram detectados anticorpos IgG anti *Toxoplasma gondii* em 40,79% (93/228) das amostras; destes, 23,6% (22/93) apresentaram altos títulos de anticorpos IgG e tiveram outras duas amostras de sangue periférico coletadas, sendo verificada a diminuição desses títulos.

**Conclusão:**

Tais achados demonstraram a importância do fortalecimento de ações junto à atenção primária à saúde para a prevenção da infecção, assim como a capacitação de profissionais da saúde que atuam nesta área, para que sejam munidos de informações referentes a casos de reinfecção e reativação da infecção em gestantes, minimizando os riscos para os bebês.

## INTRODUÇÃO

As doenças infecciosas na gestação são um dos grandes problemas enfrentados pela saúde pública. Estas incluem a toxoplasmose, rubéola, hepatites B e C, sífilis, vírus da imunodeficiência humana (HIV), entre outras, que podem ser transmitidas de mãe para filho, por via transplacentária, no momento do parto ou durante a amamentação, podendo também ocasionar aborto e parto prematuro^(1,2)^.

A assistência pré-natal deve ser feita baseada em evidências científicas e respeitando as individualidades de cada gestante, para que, assim, sejam minimizados os riscos que podem vir a surgir na gestação devido às doenças infecciosas^(3)^. Dessa forma, o Programa Nacional de Triagem Neonatal (PNTN) foi implantado no Brasil através da portaria nº 822, de 06 de junho de 2001, sendo instituído no âmbito do Sistema Único de Saúde (SUS). Esse programa consiste em um conjunto de ações que visam à prevenção e a identificação e tratamento precoce de crianças que possam apresentar doenças metabólicas, genéticas, enzimáticas e endocrinológicas^(4)^.

Dentre as principais ações do PNTN, encontra-se a realização do teste de Guthrie, conhecido como teste do pezinho, que possibilita o diagnóstico precoce de seis doenças: anemia falciforme, fenilcetonúria, hipotireoidismo congênito, deficiência de biotinidase, fibrose cística e hiperplasia adrenal congênita. Estas podem não manifestar sintomas na primeira infância, mas acarretam diversos problemas para a saúde da criança. Em 2021 o PNTN foi aperfeiçoado para estabelecer um rol de doenças a serem rastreadas pelos testes do pezinho, que será implantado de forma escalonada em cinco etapas^(5)^. O teste deve ser realizado entre o 3º e o 5º dia de vida do recém-nascido. Orienta-se que não seja realizado antes de 48h de vida e, em caso de não ter sido feito até o 7º dia de vida, ser realizado em até 30 dias após o nascimento^(6)^.

Para o risco gestacional, destaca-se a infecção por *Toxoplasma gondii*, agente etiológico da toxoplasmose, uma infecção autolimitada em pessoas saudáveis, mas que, para o período gestacional, pode apresentar diversos riscos^(7)^. A toxoplasmose é muito incidente em países de clima tropical, podendo ser adquirida principalmente pelos indivíduos por hábitos alimentares e de higiene inadequados com os alimentos e as mãos, além da forma congênita, quando transmitida de mãe para filho^(8,9)^.

A toxoplasmose por primo-infecção é a mais comum, sendo o primeiro contato do indivíduo com o parasito. Geralmente é assintomática em imunocompetentes; entretanto, após a infecção aguda, o parasito permanece na forma crônica, por meio de cistos teciduais, podendo manifestar-se em qualquer momento da vida do indivíduo^(10)^.

A toxoplasmose recebe notoriedade quando manifestada por mulheres em idade fértil, devido ao risco que apresenta para o feto. Como o processo gestacional pode acarretar uma deficiência imunológica em algumas mulheres, existe o risco de reativação dos cistos, gerando, assim, risco de comprometimento fetal, transmissão vertical, manifestações locais e sistêmicas no bebê, aborto espontâneo e morte fetal^(11)^.

No Brasil estima-se que cerca de 60% da população adulta já tenha sido exposta ao *T. gondii*, devido a sua extensão territorial e às diferenças socioculturais. Verificam-se diferentes índices de prevalência por região, mas são sempre identificados valores elevados; tal fato pode estar intimamente ligado aos hábitos alimentares e condições ambientais^(8,11)^. A taxa de mulheres em idade fértil expostas a esse parasito, apresentando infecção crônica, é de 83%^(7)^.

Quanto à toxoplasmose congênita em nível mundial, estima-se que, de cada 10 mil nascidos vivos, 1 a 10 crianças serão infectadas pelo *T. gondii* ^(8,11)^. Nos EUA, estima-se que cerca de 400 crianças nascem com toxoplasmose congênita por ano, podendo chegar a 4.000 por cada 10 mil nascidos vivos^(7)^. Na América do Sul apresenta-se uma alta taxa de incidência, sendo que no Brasil a estimativa feita pelo Ministério da Saúde (MS) é que, de cada 10 mil nascidos vivos, 3 a 20 nascerão com a toxoplasmose congênita, podendo manifestar os sinais e sintomas dessa infecção ao longo da vida^(6)^

As maiores taxas de prevalência das infecções gestacionais são identificadas em mulheres de baixa renda, o que evidencia os percalços existentes na atenção básica e no fortalecimento das ações de prevenção, diagnóstico e tratamento precoces nessa população^(12)^. As gestantes, quando expostas ao *T. gondii*, seja na infecção aguda ou na reativação de uma infecção crônica, possuem alto risco de transmissão transplacentária, podendo desencadear aborto espontâneo e sequelas fetais como patologias neurológicas e oculares, que podem vir a manifestar-se também em alterações na vida adulta.

Os profissionais de saúde responsáveis pelo acompanhamento pré-natal têm um papel muito importante na disseminação de informações e orientações de qualidade a respeito da doença, visando à minimização dos fatores de risco, por parte das gestantes. Esses profissionais também devem realizar a solicitação dos exames laboratoriais no decorrer da gestação, além de orientar as gestantes sobre a sua importância. A partir desses exames, é possível verificar a presença do parasito e iniciar o tratamento, visando a diminuição do risco de transmissão transplacentária, além de fazer o acompanhamento do recém-nascido por meio do teste do pezinho.

Diante do exposto, são necessários estudos que evidenciem o perfil clínico laboratorial e epidemiológico da toxoplasmose congênita em recém-nascidos de modo a melhorar a qualidade das ações de prevenção, diagnóstico e tratamento. O objetivo proposto nesse estudo é realizar a triagem sorológica da toxoplasmose no teste do pezinho e avaliar os aspectos epidemiológicos em recém-nascidos e puérperas de Jataí, Goiás.

## MÉTODO

### Tipo do Estudo

Trata-se de um estudo epidemiológico transversal.

### Local

O estudo foi desenvolvido no município de Jataí, Goiás, no único local onde é realizada a triagem neonatal de recém-nascidos, e no Laboratório de Bioquímica Clínica e Líquidos Corporais do curso de Biomedicina da Universidade Federal de Jataí.

### Critérios de Seleção

As amostras foram selecionadas por conveniência, por meio dos pais e/ou responsáveis que levavam os recém-nascidos à PMS para a realização do teste do pezinho entre o terceiro e o sétimo dia de vida. Estes eram informados pelos pesquisadores sobre a pesquisa e convidados a participar. Após a leitura e assinatura do Termo de Consentimento Livre e Esclarecido (TCLE), foi realizada a coleta da amostra de papel filtro. Os pesquisadores responsáveis são biomédicos e enfermeira.

Quando os recém-nascidos, após a análise do ELISA, apresentaram resultado IgM positivo ou altos títulos de anticorpos IgG, os seus pais e/ou responsáveis foram comunicados e foi solicitada a autorização para a segunda coleta de uma amostra de sangue periférico do bebê e da mãe para a realização de novos testes laboratoriais, ELISA, para que fossem acompanhados e monitorizados pelos pesquisadores, visando à identificação da toxoplasmose congênita. Esta coleta foi realizada no domicílio dos participantes.

### Definição da Amostra

Participaram desta pesquisa 228 recém-nascidos. Foi calculada a média de testes do pezinho realizados no município de Jataí no período que antecedeu a pandemia de COVID-19. No ano de 2017 foram realizadas 1.216 coletas de teste do pezinho; no ano de 2018, 1.327 coletas; no ano de 2019, 1.248 coletas; e no ano de 2020, 1.104 coletas. A partir disso, realizou-se o cálculo amostral, sendo necessário uma quantidade mínima de 205 amostras de teste do pezinho para a realização deste estudo, que poderia apresentar uma margem de erro de 6,25%^(13)^.

### Coleta de Amostras Biológicas em Papel Filtro

O período de coleta das amostras de sangue em papel filtro dos recém-nascidos foi compreendido entre os meses de setembro de 2021 a maio de 2022.

Os RNs cujos pais permitiram a coleta tiveram a amostra de sangue coletada em papel filtro Whatman nº 1, de 3 cm. As amostras foram acondicionadas em prateleiras na posição horizontal, de modo que não houvesse o contato com as demais, até que ele estivesse seco (temperatura ambiente). Então, foram guardados em envelopes adequados e identificados por meio de carimbo padronizado. O material foi transportado para o Laboratório de Bioquímica Clínica e Líquidos Corporais para análises sorológicas, sendo que o acondicionamento e o transporte seguiram as recomendações do Manual Técnico de Triagem Neonatal^(6)^.

### Padronização da Técnica de Enzimaimunoensaio em Papel Filtro

Realizou-se a padronização da técnica e definição de títulos, conforme as orientações fornecidas pelo fabricante do kit comercial *SERION ELISA classic-Toxoplasma gondii IgG/IgM* ^®^. Após avaliação dos resultados seguindo o determinado pelo “*Quality Control Certificate*”, definiram-se como alto título de anticorpos IgG os valores acima de DO 1,4494 na análise de absorbância.

### Preparo das Amostras e Realização do Diagnóstico Sorológico

As amostras de papel filtro foram preparadas no dia anterior à realização do teste, sendo necessários os equipamentos de proteção individual (EPI), um perfurador individualizado para picotar círculos de 3 a 5 mm, tubo tipo *eppendorf* e pipeta.

Para a dosagem de IgM, o círculo foi eluído em uma solução de RF-*absorbent* ¼, sendo, então, 1 círculo de 3 a 5 µL + 100 µL de RF-*absorbent* + 400 µL de tampão de amostra. Para a detecção de IgG, a amostra foi eluída em 100 µL de tampão de amostra. Para ambas as dosagens, o papel filtro foi completamente coberto com o tampão, não podendo flutuar dentro do tubo, sendo então incubados *overnight* na geladeira de 2 a 8ºC.

As amostras foram então submetidas ao exame sorológico e, a partir desse resultado, foi possível verificar a presença do *T. gondii* e as imunoglobulinas IgM e IgG.

### Acompanhamento dos Recém-Nascido com Altos Títulos de Anticorpos

Os bebês que apresentaram altos títulos de anticorpos IgG (DO ≥ 1,4494) na análise de absorbância foram acompanhados a fim de verificar possíveis manifestações da infecção congênita de toxoplasmose, assim como seus dados laboratoriais, sendo então acompanhados um total de 22 bebês.

A segunda coleta, venosa, foi realizada com margem de segurança de três meses da coleta inicial em papel filtro, levando em consideração a meia vida dos anticorpos IgM e IgG, permitindo assim que fossem verificados os anticorpos do bebê e não da mãe. Uma amostra de 5 mL sangue periférico foi coletado da mãe e do bebê, acondicionada em tubos para obtenção de soro e transportadas ao Laboratório de Bioquímica Clínica e Líquidos corporais da UFJ.

Após o período de cerca de um ano, foi feita uma nova coleta de sangue dos bebês acompanhados, a fim de verificar se os títulos de anticorpos se mantinham em diminuição ou se estavam não reagentes.

Obteve-se autorização para a realização da terceira coleta venosa de 18 crianças; porém, os pais e responsáveis foram orientados acerca das manifestações clínicas da toxoplasmose congênita e adquirida, assim como sobre os serviços de referência no município de Jataí, para a atenção à saúde da criança.

### Aplicação do Questionário

Quando foi realizada a coleta da segunda amostra de sangue do bebê, também foi realizada uma coleta de sangue da mãe. Além disso, foi aplicado um questionário socioeconômico e comportamental construído pelos próprios pesquisadores, de modo a contemplar as informações relevantes sobre o perfil da população estudada, a 22 mães dos bebês acompanhados devido aos altos títulos de anticorpos. As mães que aceitaram participar da pesquisa foram entrevistadas em local apropriado, seguindo todas as recomendações de segurança e protocolos de saúde propostos pelo serviço de saúde. Os dados coletados foram utilizados para análise epidemiológica como fatores de risco para contaminação/transmissão do *T. gondii*. Nesse momento, foi feita a avaliação do recém-nascido, a fim de verificar possíveis alterações causadas pela toxoplasmose, para o seguimento do acompanhamento, e foi preenchido o documento norteador para visita domiciliar.

### Análise e Tratamento dos Dados

Os dados foram analisados a partir do programa Microsoft Excel^®^ 2021 e apresentados na forma de estatística analítica descritiva, de modo a evidenciar a prevalência de gestantes com toxoplasmose com possível infecção congênita do recém-nascido.

### Aspectos Éticos

O projeto foi aprovado pelo Comitê de Ética em Pesquisa (CEP), com parecer número 4.925.393, no ano de 2021. Todos os participantes assinaram o TCLE para participar e foram seguidas todas as orientações constantes nas Resoluções nº 466/2012 e nº 510/2016, que tratam da pesquisa com seres humanos.

## RESULTADOS

### Triagem Sorológica em Papel Filtro

Das 228 amostras analisadas, foram detectados anticorpos IgG anti-*T. gondii* em 40,8% (93/228), não sendo identificado IgM pelo teste ELISA. Das amostras positivas para o anticorpo IgG para *T. gondii*, 23,6% (22/93) apresentaram valores elevados de anticorpos, sendo encaminhados para uma nova coleta de amostra de sangue periférico do bebê e da mãe. Na Figura 1 observa-se o quantitativo de amostras avaliadas neste estudo.

**Figura 1:** Distribuição do número de amostras.

Realizou-se um comparativo das sorologias dos bebês na amostra inicial e segunda e terceiras coletas, com suas respectivas mães, observado na Tabela 1.

**Tabela 1:** Comparativo de sorologias Imunoglobulina G coletadas entre setembro de 2021 a maio de 2022 e em abril de 2023 – Jataí, GO, Brasil, 2023.

Código	1ª coleta* Papel Filtro		2ª coleta** Sangue periférico				3ª coleta*** Sangue periférico	
	Criança		Mãe		Criança		Criança	
002	1,491	POS	2,175	POS	0,241	NEG	0,023	NEG
003	1,577	POS	2,023	POS	0,246	NEG	0,028	NEG
006	1617	POS	2,258	POS	0,210	NEG	0,008	NEG
056	1,427	POS	2,214	POS	0,162	NEG	0,056	NEG
091	1,451	POS	2,124	POS	0,204	NEG	0,014	NEG
151	1,499	POS	2,395	POS	0,175	NEG	0,043	NEG
174	1,633	POS	3,020	POS	0,243	NEG	0,025	NEG
177	1,654	POS	2,731	POS	0,305	NEG	0,087	NEG
179	1,464	POS	2,086	POS	0,321	NEG	0,103	NEG
182	1,488	POS	2,149	POS	0,297	NEG	0,079	NEG
183	1,553	POS	2,174	POS	0,181	NEG	0,037	NEG
186	1,642	POS	2,985	POS	0,249	NEG	0,031	NEG
208	1,862	POS	2,325	POS	0,229	NEG	0,011	NEG
209	2,148	POS	3,122	POS	0,235	NEG	NR	NEG
210	2,240	POS	2,996	POS	0,222	NEG	0,017	NEG
211	1,951	POS	2,827	POS	0,292	NEG	NR	NEG
214	1,582	POS	1,795	POS	0,195	NEG	0,004	NEG
223	1,458	POS	1,637	POS	0,302	NEG	0,074	NEG
224	2,073	POS	2,894	POS	0,241	NEG	NR	NEG
225	1,875	POS	3,061	POS	0,288	NEG	NR	NEG
226	1,890	POS	3,038	POS	0,232	NEG	0,023	NEG
228	1,862	POS	3,073	POS	0,323	NEG	0,084	NEG

*Coleta de papel filtro, realizada com 03 a 05 dias de vida. ** Coleta com 3 a 4 meses. *** Coleta com 12 a 18 meses. Legenda: POS: positivo, NEG: negativo.

### Perfil Sociodemográfico

Foi verificada uma média de idade de 24,3 anos (18 anos – 30 anos), a maioria das participantes são casadas, 72,7% (16/22), todas residentes no município de Jataí-GO, com residência na zona urbana. A escolaridade mais frequente foi o ensino médio completo: 81,8% (18/22). No que tange ao saneamento básico e hábitos de vida dos pais entrevistados, os achados podem ser observados no quadro abaixo (Quadro 1).

**Quadro 1:** Perfil sociodemográfico dos responsáveis dos participantes do estudo – Jataí, GO, Brasil.

Variáveis		Nº (n = 22)	Percentual (%)
Origem da água de consumo	Rede pública	22	100
Destino do esgoto	Rede pública	22	100
Destino do lixo domiciliar	Coleta pública	22	100
Horta em casa	Sim	2	9,1
	Não	22	90,9
Pessoa que prepara o alimento	Mãe	17	77,28
	Outra pessoa	5	22,72
Possui animais em casa	Gato	0	0
	Cachorro	6	27,8
Hábitos comportamentais e alimentares	Manuseia terra ou areia	2	9,1
	Consome carne diariamente	22	100
	Consome carne crua ou mal-cozida esporadicamente	5	22,7

Quando levada em consideração a análise do perfil sociodemográfico, percebeu-se que as mulheres estavam, em média, com duas gestações. Apenas uma entrevistada relatou um caso de aborto sem causa definida. A idade gestacional média no momento do parto foi de 39 semanas (37 – 41 semanas). Todas as entrevistadas fizeram o pré-natal pelo Sistema Único de Saúde (SUS) em Unidades Básicas de Saúde mais próximas ao domicílio, tendo todas as mulheres realizado o teste da mamãe nas duas etapas, conforme conferido no cartão da gestante. Os resultados foram reagentes para IgG anti *T. gondii* e nenhuma apresentou anticorpos IgM reagentes. Todas tiveram gestações de risco habitual.

## DISCUSSÃO

As amostras de recoletas analisadas pelo método ELISA demonstraram uma diminuição significativa dos valores de anticorpos IgG nas crianças, o que permite inferir que os anticorpos da primeira sejam provenientes das mães, tendo atravessado passivamente a barreira transplacentária e passado a circular no sangue do bebê^(8,9)^.

A alta prevalência de mulheres cronicamente infectadas na região, verificada pelas altas taxas de bebês com a circulação de anticorpos para *T. gondii*, é um fator preocupante, pois aumenta os riscos de reativação da infecção nas mulheres e possível transmissão congênita^(11)^. Além disso, observa-se que pode haver o risco aumentado de infecção das mulheres em idade fértil, devido à alta circulação do parasita no município analisado^(13)^.

Em comparação com outras regiões do Brasil, verifica-se que a prevalência de toxoplasmose gestacional no município corrobora o achado em Goiânia, 41,8%^(14,15)^, em Santa Catarina, 47%^(15)^, em Sergipe, com aproximadamente 50%^(16)^, em São Paulo, 54,91%^(17)^, e que o município apresenta uma menor prevalência quando comparado aos achados clínicos no Amapá, com 73,43% de gestantes infectadas^(18)^, e maior se comparado ao estado do Rio Grande do Norte, com uma prevalência de 24,4%^(19)^. Esses dados reforçam que o Brasil é um país que apresenta altos índices de infecção por *T. gondii*, principalmente em mulheres em idade fértil, o que valida a importância de ações de vigilância em saúde, visando a minimização de riscos de infecções congênitas.

Neste estudo, não foram encontrados casos de toxoplasmose congênita, que, apesar de ser uma forma grave da doença, é menos comum de ocorrer. Em estudo de coorte com mulheres rastreadas no início da gravidez, foi verificado que a taxa de transmissão vertical é < 5% se a infecção da mulher ocorrer no período de 3 meses que antecede a gestação ou nos primeiros meses, podendo chegar a 71% se houver infecção aguda após as 37 semanas de gravidez^(20)^. Em países como os Estados Unidos, nos anos de 2006 a 2014, foi verificado que a incidência de toxoplasmose congênita foi de apenas 0,23% por 10 mil nascidos vivos^(21)^.

O pré-natal feito no SUS, sendo este o formato escolhido pelas mulheres entrevistadas neste estudo, apresenta desigualdades regionais, mas possui cobertura elevada em todo o país. Na região centro-oeste, além da alta cobertura, também é verificado que as mulheres iniciam precocemente esse acompanhamento, o que permite uma melhor avaliação e mais tempo para que sejam repassadas informações importantes sobre a gestação^(22)^.

Entretanto, é verificado que, apesar da alta adesão ao pré-natal, são baixos os índices de pré-natais considerados adequados, quando levados em consideração aqueles que são realizados contemplando o mínimo de seis consultas pré-natal, realização de todos os exames laboratoriais necessários e de no mínimo três ultrassonografias de acompanhamento, além da realização de orientações verídicas, pautadas no conhecimento técnico e científico transmitido de forma clara e objetiva, para que seja compreendido na sua totalidade, sendo que o não cumprimento desses requisitos piora os desfechos de saúde de gestantes que necessitam de atenção^(23)^.

Quanto à orientação dos profissionais de saúde, 86,4% (19/22) relataram terem sido informadas sobre a toxoplasmose quando foi entregue o resultado do exame, mas referiram que foi uma orientação superficial, não sendo repassadas informações importantes quanto ao achado de anticorpos anti-IgG para *T. gondii*. Outras 13,63% (3/22) não tiveram nenhum tipo de orientação sobre os achados nos exames de teste da mamãe.

Em consonância com o que foi encontrado em outros estudos, os profissionais de saúde não apresentam conhecimentos adequados sobre a toxoplasmose, principalmente sobre a forma congênita da doença, casos de reinfecção e reativação da infecção por *T. gondii*, assim como sobre os riscos de transmissão, medidas profiláticas e manifestações clínicas nos bebês^(12,24)^. Além disso, muitos profissionais consideram que o aparecimento de anticorpos IgG, nos exames pré-natais, seja um fator de proteção para a gestante. Assim, não são repassadas informações importantes para elas sobre a suscetibilidade de reinfecção e reativação do protozoário, medidas profiláticas eficazes e sinais e sintomas da doença^(12)^.

A falta de conhecimento dos profissionais de saúde leva-os a negligenciar os casos de toxoplasmose gestacional, aumentando assim o risco de transmissão transplacentária e o desenvolvimento de toxoplasmose congênita nos bebês, bem como expondo as mulheres e crianças a outros riscos de manifestações da doença^(11)^. Ressalta-se que, quando o profissional de saúde identifica corretamente uma infecção aguda pelo *T. gondii* e inicia precoce e corretamente o tratamento das gestantes, as chances de transmissão vertical são reduzidas drasticamente, principalmente se iniciado antes da terceira semana após a soroconversão^(25)^.

Em países como a Áustria e a França, já foi possível observar uma diminuição da prevalência de toxoplasmose e incidência de infecções congênitas por meio da implantação da triagem pré-natal, favorecendo o início precoce do tratamento, assim como o fortalecimento das ações de educação em saúde^(26)^. No Brasil, a triagem pré-natal é regulamentada pelo Ministério da Saúde, com orientações por meio de protocolos e manuais, visando à detecção precoce de infecções prévias que podem ocorrer ao longo da gravidez^(27)^. No Estado de Goiás, todas as gestantes que realizam o acompanhamento pré-natal no SUS realizam o teste da mamãe, que contempla o exame de toxoplasmose^(28)^. Sendo assim, é extremamente importante que os profissionais de saúde tenham o conhecimento para a interpretação dos exames e realização de orientações corretas, uma vez que o acesso da mulher aos exames é garantido^(12,27)^.

As limitações do presente estudo foram relacionadas principalmente à dificuldade de abordagem das famílias para as etapas de acompanhamento dos bebês, visto que muitas, por desconhecer a doença e serem pouco ou erroneamente orientadas nos serviços de saúde, não reconheciam a necessidade do procedimento.

As contribuições deste estudo foram principalmente para o conhecimento do perfil epidemiológico do município, além do levantamento de informações sobre a qualidade das orientações repassadas às famílias sobre a toxoplasmose, pelos profissionais de saúde, sendo verificada a necessidade de atualizá-los para que reconheçam a importância da doença, os cuidados no ciclo gravídico, a interpretação correta de exames e a existência de casos como reinfecção e reativação da infecção.

## CONCLUSÃO

Verificou-se que a triagem neonatal para toxoplasmose permitiu a detecção de anticorpos IgG, sendo este elevado no município, pois foram encontrados 93 casos, apresentando assim um considerável índice de infecções crônicas em mulheres em idade fértil. Tais achados demonstram a importância do fortalecimento de ações junto à atenção primária à saúde para a prevenção da infecção, assim como a capacitação de profissionais da saúde que atuam nesta área, para que sejam munidos de informações referentes a casos de reinfecção e reativação da infecção em gestantes, de modo a minimizar os riscos para os bebês.
